# *In vivo* distribution of U87MG cells injected into the lateral ventricle of rats with spinal cord injury

**DOI:** 10.1371/journal.pone.0202307

**Published:** 2018-08-16

**Authors:** Jeong-Seob Won, Hyun Nam, Hye Won Lee, Ji-Yoon Hwang, Yu-Jeong Noh, Do-Hyun Nam, Sun-Ho Lee, Kyeung Min Joo

**Affiliations:** 1 Department of Health Sciences and Technology, SAIHST, Sungkyunkwan University, Seoul, South Korea; 2 Single Cell Network Research Center, Sungkyunkwan University School of Medicine, Suwon, South Korea; 3 Stem Cell and Regenerative Medicine Center, Research Institute for Future Medicine, Samsung Medical Center, Seoul, South Korea; 4 Department of Neurosurgery, Samsung Medical Center, Sungkyunkwan University School of Medicine, Seoul, South Korea; 5 Department of Anatomy & Cell Biology, Sungkyunkwan University School of Medicine, Suwon, South Korea; University of Utah Hospital, UNITED STATES

## Abstract

Stem cells could be the next generation therapeutic option for neurodegenerative diseases including spinal cord injury (SCI). However, several critical factors such as delivery method should be determined before their clinical applications. Previously, we have demonstrated that lateral ventricle (LV) injection as preclinical simulation could be used for intrathecal administration in clinical trials using rodent animal models. In this study, we further analyzed *in vivo* distribution of cells that were injected into LVs of rats with SCI at thoracic level using *in vivo* imaging techniques. When 5 × 10^6^ U87MG cells labelled with fluorescent magnetic nanoparticle (FMNP-labelled U87MG) were administrated into LVs at 7 days after SCI, FMNP-labelled U87MG cells were observed in all regions of the spinal cord at 24 hours after the injection. Compared to water-soluble Cy5.5 fluorescent dye or rats without SCI, *in vivo* distribution pattern of FMNP-labelled U87MG cells was not different, although migration to the spinal cord was significantly reduced in both Cy5.5 fluorescent dye and FMNP-labelled U87MG cells caused by the injury. The presence of FMNP-labelled U87MG cells in the spinal cord was confirmed by quantitative PCR for human specific sequence and immunohistochemistry staining using antibody against human specific antigen. These data indicate that LV injection could recapitulate intrathecal administration of stem cells for SCI patients. Results of this study might be applied further to the planning of optimal preclinical and clinical trials of stem cell therapeutics for SCI.

## Introduction

Spinal cord injury (SCI) is a devastating condition that causes substantial morbidity and mortality [1]. Since no effective treatment modalities for SCI are currently available, transplantation of stem cells has been developed as an alternative treatment. Stem cells have regenerative potentials that can repopulate damaged neural cells in the injured neural tissue of SCI with paracrine effects that can help damaged neural cells survive [2]. However, several critical factors such as clinical delivery route of stem cells, stem cell viability after transplantation, and *in vivo* stem cell migration capacity still remain unclear. They should be clearly accounted for prior to their clinical applications. These factors can significantly affect the safety and treatment results of stem cells [3, 4]. Therefore, preclinical animal experiments addressing those issues are essential.

There are several candidate routes for administration of stem cells into SCI patients. In preclinical studies, direct injection of stem cells into damaged spinal cord regions is commonly used [5, 6]. However, this route is hard to be translated to clinical trials since it might induce secondary injuries to the spinal cord [7]. Instead, intrathecal injection of stem cells has been considered in clinical trials, expecting stem cells to migrate into disease sites via cerebrospinal fluid (CSF) [8–10]. To simulating clinical situation in animal models, we have injected Cy5.5 fluorescent dye into the lateral ventricle (LV) or cisterna magna (CM) of rat without SCI and compared its *in vivo* distribution in each region of spinal cord [11]. LV injection is more suitable than CM injection since it induces widespread distribution of Cy5.5 in spinal cords [11].

However, there are many differences in *in vivo* distribution characteristics between soluble fluorescent dye and colloidal stem cells. Therefore, it is necessary to determine *in vivo* distribution of cells. Moreover, SCI could affect the distribution of materials in CSF. To address these subjects further, we injected Cy5.5 fluorescent dye or cells labelled with fluorescent magnetic nanoparticles (FMNPs) into LVs of rats with or without SCI in this study and analyzed their *in vivo* distributions using *in vivo* optical imaging techniques. The localization of FMNP-labelled cells in each region of spinal cord was validated further by quantitative PCR and immunohistochemistry staining.

## Materials and methods

### Animal care

This study was reviewed and approved by the Institutional Animal Care and Use Committee (IACUC) of Samsung Biomedical Research Institute (SBRI, Seoul, South Korea) (approval number: 20160719001). SBRI is an Association for Assessment and Accreditation of Laboratory Animal Care International (AAALAC International) accredited facility that abides by the Institute of Laboratory Animal Resources (ILAR) guide. Animal experiments were conducted in accordance with the Institute for Laboratory Animal Research Guide for the Care and Use of Laboratory Animals following protocols approved by the Institutional Review Board (IRB) at the Samsung Medical Center (SMC, Seoul, South Korea) (approval number: 2014-03-014-006). All rats were sacrificed in a CO_2_ chamber.

### Cell culture and labeling with fluorescent magnetic nanoparticle (FMNP)

Human glioblastoma U87MG cells were cultured in Dulbecco’s modified Eagle’s medium (DMEM)-high glucose (Welgene, Gyeongsan, South Korea) supplemented with 10% v/v fetal bovine serum (FBS, Gibco, Grand Island, NY, USA) and 100 U/ml penicillin/streptomycin (P/S, Gibco). For labeling with FMNP, U87MG cells were cultured to 90% confluency. Rhodamine isothiocyanate (RITC) was conjugated to terminal silanol groups of FMNP (50 nm in size, NEO-STEM^™^ TSR50, Biterials Co., Ltd, Seoul, South Korea). Labelling with FMNP was performed according to the instruction of the manufacturer. Briefly, 2 mg/ml FMNP in 1 ml borate buffer solution was added to 50 ml culture medium. FMNP was precipitated by ultracentrifugation at 12,000 rpm for 10 minutes, diluted to final concentration of 0.2 mg/ml in culture medium, and then dispersed by strong ultrasonication (Sonic Dismembrator Model 500, Fisher Scientific, Hampton, NH) at 300 Watt for 5 minutes at room temperature (RT). U87MG cells were incubated with the medium containing 0.2 mg/ml FMNP for 24 hours in a 37°C incubator with 5% CO_2_. After incubation, the medium was replaced with fresh culture medium. Labeling of U87MG cells with FMNP was confirmed with a fluorescence microscope (Axio observer Z1, Zeiss, Jena, Germany).

### Spinal cord injury (SCI) modeling

Under isoflurane (Ifran^™^, Hana Pharm, Seoul, South Korea) anesthesia, thoracic spinal cords (T8-10) of adult female Sprague-Dawley rats (10-week-old, body weight of 250–300 g, Orient Bio., Sungnam, South Korea) were exposed by laminectomy. Using a Multicenter Animal Spinal Cord Injury Study (MASCIS) impactor (Rutgers University, New Brunswick, NJ, USA), a 10 g rod was dropped from a vertical distance of 25 mm onto the T9 level spinal cord. After contusion, the muscle, subcutaneous layer, and skin were sutured according to their respective anatomical layers. All animals received an intramuscular injection of 10 mg/kg ketoprofen (Uni Biotech, Seoul, South Korea) to reduce pain after the surgery. Motor function of hind limbs after SCI was evaluated using Basso-Beatti-Bresnahan (BBB) locomotor rating test on open field [12]. Bladder compression was performed once daily until rats were sacrificed.

### Lateral ventricle injection

Rats with SCI received lateral ventricle injection at seven days after SCI. Lateral ventricle injection was performed according to a previous report [13]. Briefly, under isoflurane anesthesia, rats were fixed in a stereotaxic device (Model 900 small animal stereotaxic instrument, KOPF stereotaxic, Tujunga, CA, USA). The skin on skull was cut about 2 cm and the external membrane was removed to expose the bregma. The injection site [Medial/Lateral (ML): 1.2 mm, Anterior/Posterior (AP): -0.3 mm, Dorsal/Ventral (DV): 4 mm from the bregma] was marked with a Hamilton syringe (26G, Hamilton Company, Reno, NV, USA) connected to a stereotaxic device and a 0.5 mm diameter hole was made using a drill (8050-N/18 micro 8V max, Dremel, Racine, WI, USA). Then 20 nM Cy5.5 fluorescent dye (Amersham CyDye^™^ mono-reactive NHS Ester, GE Healthcare, Piscataway, NJ, USA) or 5 × 10^6^ U87MG cells in 50 μL HBSS (Gibco) was injected into the lateral ventricle (4 mm deep from the skull) using a syringe pump (LEGATO^™^111, KD scientific, Holliston, MA, USA) for 10 minutes. After injection, the injection needle was maintained for 5 minutes to prevent leakage. The injection needle was then raised up carefully at a speed of 1 mm per minute. All animals were administrated with 10 mg/kg ketoprofen to reduce pain after the surgery.

### *In vivo* and *ex vivo* near-infrared fluorescence (NIRF) imaging

Optical NIRF imaging was conducted at 24 hours after the injection of Cy5.5 fluorescent dye or U87MG cells labeled with FMNP using a Xenogen IVIS Spectrum system (Caliper Life Science, Hopkinton, MA, USA) according to previous reports [14, 15]. Identical illumination settings (lamp voltage, filters, f/stop, field of views, and binning) were used for all images. Fluorescence (emission: 720 nm, excitation: 605 nm for Cy5.5 fluorescent dye, emission: 581 nm, excitation: 558 nm for FMNP) was measured as photons per second per centimeter squared per steradian (p/s/cm^2^/sr). Quantitative data in each region of interest (ROI) were acquired and analyzed using Living Image 2.5 software (Caliper Life Science). The head, cervical, thoracic, and lumbar regions were defined from eyes to the first cervical vertebrae (C1), from C1 to the first thoracic vertebrae (T1), from T1 to the first lumbar vertebrae (L1), and from L1 to the first sacral vertebrae (S1), respectively. In *ex vivo* analysis, the head was subdivided further into the head and cisterna magna (CM) region defined as from the forebrain to the cerebellum and from the cerebellum to the C1, respectively. Total flux was measured at each region and the sum of total fluxes at all regions was then obtained. The distribution ratio in each region was the ratio: the total flux of the region divided by the sum of total fluxes.

### Quantitative real-time PCR (qPCR)

Each region of the central nervous system (CNS) was sampled at 24 hours after lateral ventricle injection. Injection of HBSS was used as negative control (media injection group). Genomic DNA (gDNA) was extracted using a DNeasy^®^ Blood and Tissue Kit (250) (Qiagen, Hilden, Germany). Alu PCR primers (forward = 5′- TCAGGAGATCGAG-ACCATCCC-3′; reverse = 5′-TCCTGCCTCAGCCTCCCAAG-3′) [16] and 5× HOT FIREPol^®^ EvaGreen^®^ qPCR Mix Plus (Solis BioDyne, Tartu, Estonia) were used. Reaction mixture (20 μl) contained 4 μl 5 × HOT qPCR Mix, 3 μl primers (3 pM), 2 μl gDNA (5 ng), and 12.5 μl distilled H_2_O. Annealing temperature was 68°C. The expression level of Alu genes was determined by method of 2^-ΔCT^. Arbitrary Unit represented 2^-ΔCT^ × 10^11^ values. For visualization, qPCR products (5 μl) were separated on 2% agarose gels.

### Immunohistochemistry staining

Brain and spinal cord were removed and embedded in paraffin as described previously [17]. Paraffin blocks were sectioned (4 μm in thickness) using a microtome (Leica, Wetzlar, Germany). These sections were put on silane-coated microscope slides (Muto pure Chemicals Co., Ltd., Tokyo, Japan). Slides were heated on a slide warmer (Lab-line Instruments USA, Dubuque, IA, USA) at 65°C for 30 minutes. For antigen retrieval, deparaffinized and rehydrated sections were incubated with target retrieval solution (Dako, Carpentaria, CA) at 125°C for 30 minutes. These slides were then incubated in 3% hydrogen peroxide in methanol for 12 minutes to quench endogenous peroxidase activities. Primary anti-human cytoplasm antibody (STEM121^™^, 1:500, StemCells, Inc., Newark, CA, USA) as incubated with slides at 4°C overnight. Slides were then incubated with biotinylated secondary antibody (Vector Laboratories Inc., Burlingame, CA, USA) at RT for 1 hour followed by incubation with avidin-biotin complex (Vector Laboratories Inc.) at RT for 1 hour. These slides were then stained with DAB (3,3’-diaminobenzidine tetrahydrochloride) and counterstained with hematoxylin.

### Statistics

Data are presented as mean ± standard error. Data were analyzed using Student’s t-test, two-tailed. P-values < 0.05 were considered statistically significant.

## Results

### Spinal cord injury (SCI) modeling

SCI was induced by physical impact at T9 level of the spinal cord using a MASCIS impactor as described previous [18]. Briefly, laminectomy was performed to expose the spinal cord without disrupting the dura (S1A Fig, left). After weight free drop impact (25 mm height) by a 10 g rod (2.5 mm in diameter), SCI was obvious (S1A Fig, right). When locomotor dysfunction was measured by BBB locomotor scale [12] at 7 days after the impact, the score decreased dramatically from 21 ± 0 (normal group, mean ± SEM, n = 10) to 3.8 ± 1.03 (SCI group, n = 20) (S1A and S1C Fig).

### Quantitative in vivo distribution of Cy5.5 fluorescent dye in central nervous system (CNS) after SCI

**Fig 1:**
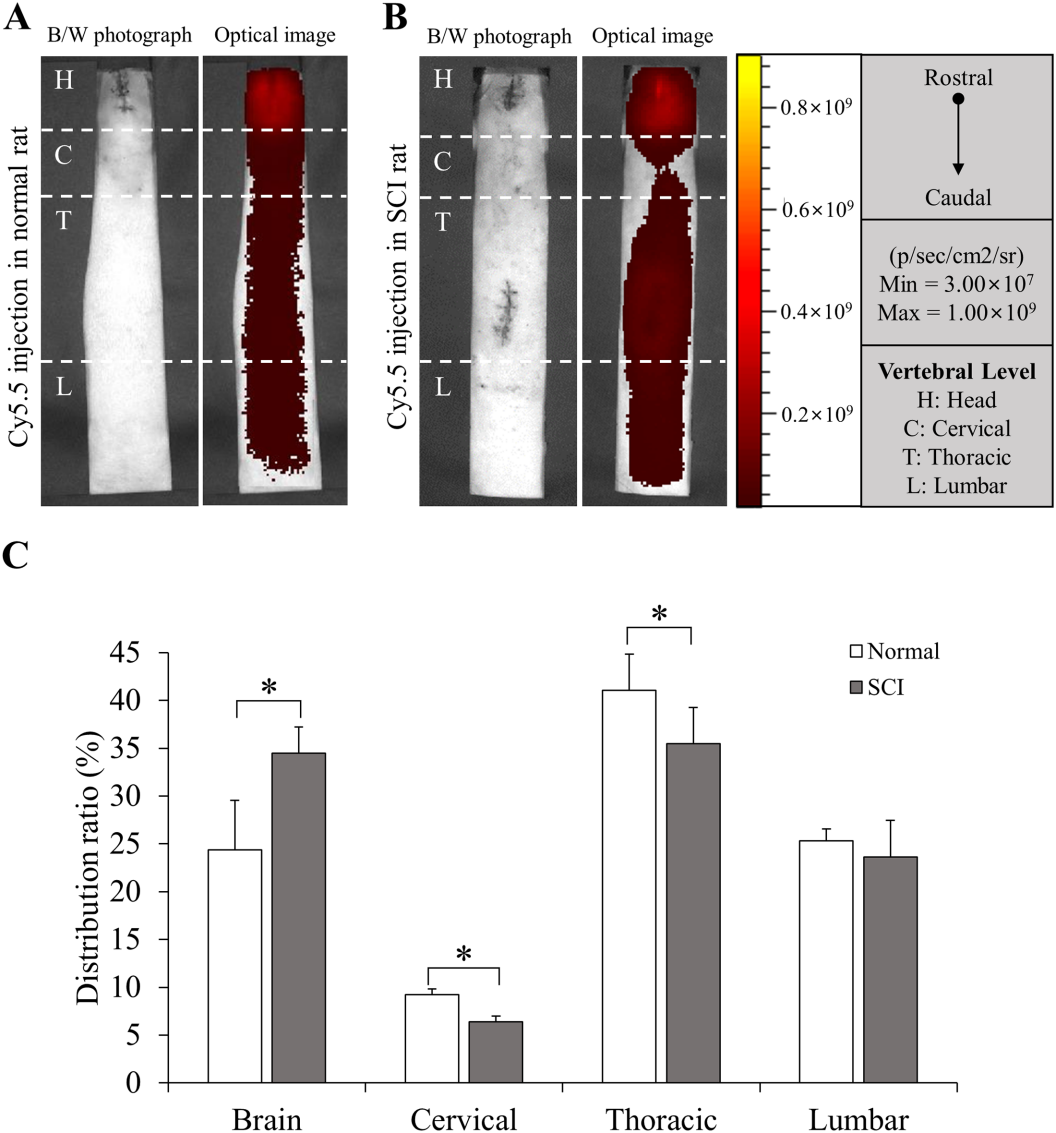
Quantitative *in vivo* distribution of Cy5.5 fluorescent dye. (A, B) The distribution of Cy5.5 fluorescent dye was determined by *in vivo* optical imaging at 24 hours after injection. The injection was performed at 7 days after SCI for rats with SCI (A for normal group, B for SCI group, n = 5 for each group). H = Head, C = Cervical, T = Thoracic, L = Lumbar. (C) The distribution ratio of Cy5.5 fluorescent dye was quantified and compared. Height = Average, Error bar = Standard deviation. *, *P* < 0.05.

We detected *in vivo* signal of Cy5.5 fluorescent dye utilizing *in vivo* optical imaging techniques in the CNS of rats with or without SCI (SCI and normal group, respectively, n = 5 for each group) at 24 hours after injection into the lateral ventricle (LV). The injection was performed at 7 days after SCI for rats with SCI. Motor dysfunction was confirmed by BBB score (S1C Fig). When the *in vivo* intensity of fluorescent signal was visualized (Fig 1A and 1B), Cy5.5 fluorescent dye was well distributed from the brain to the lumbar spinal cord in both normal and SCI groups. However, the SCI group showed significantly higher distribution ratio (%) in the brain than the normal group whereas distribution ratios (%) of the cervical and thoracic spinal cord of the SCI group were significantly lower than those of the normal group (Fig 1C). These results indicate that Cy5.5 fluorescent dye can migrate to lower spinal cord even though thoracic spinal cord is injured physically. However, its migration is suppressed by SCI.

### Quantitative *in vivo* distribution of U87MG cells labelled with FMNP in CNS after SCI

**Fig 2:**
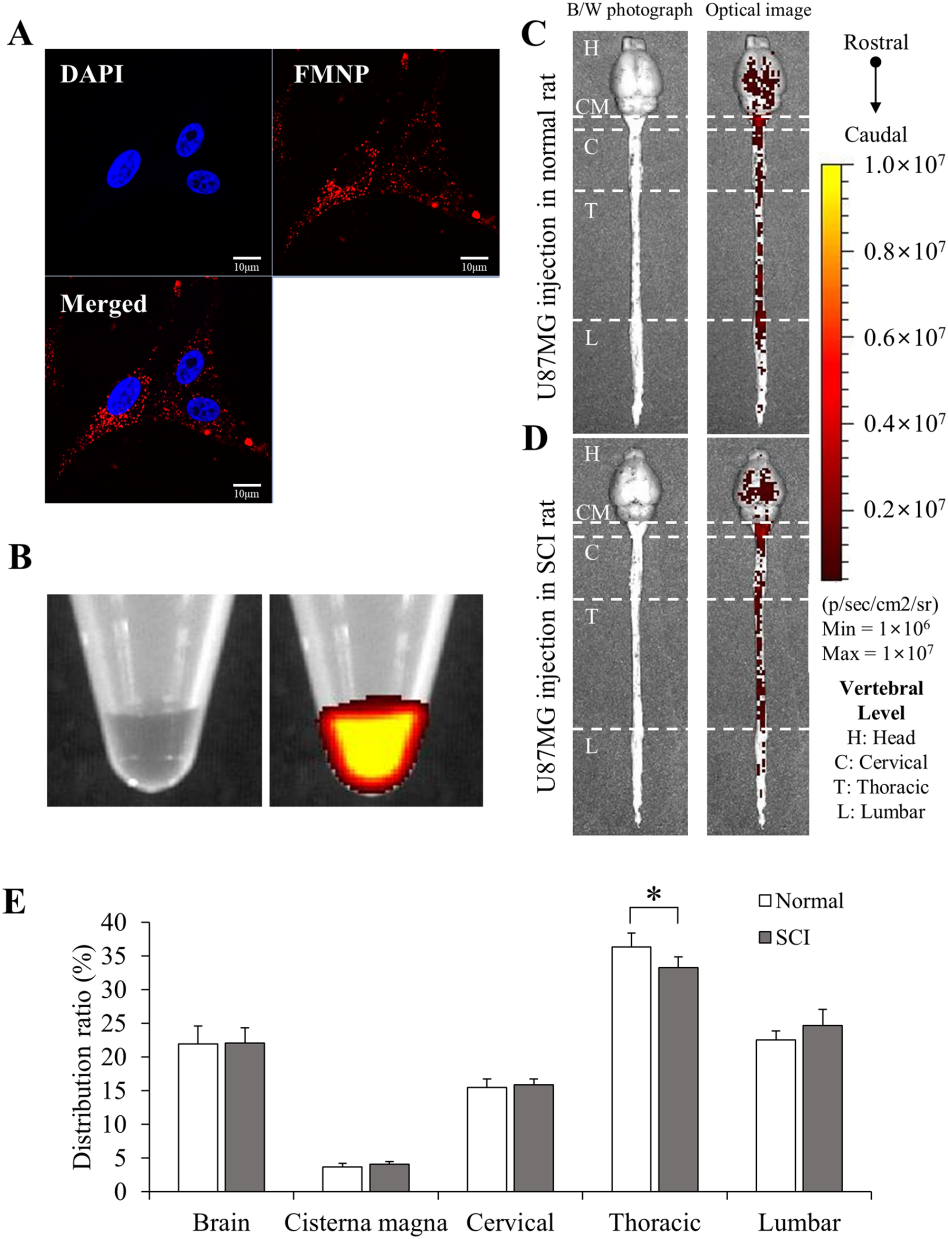
Quantitative *in vivo* distribution of U87MG cells labelled with FMNP. (A) Fluorescence (red) of FMNP-labelled U87MG cells was observed under a microscope. DAPI (blue) = nuclei. Scale bar = 10 μm. (B) Fluorescent signals of pellets of 5 × 10^6^ U87MG (left) and FMNP-labelled U87MG (right) were detected by optical imaging. (C and D) 5 × 10^6^ FMNP-labelled U87MG cells in 50 μl HBSS were injected into LVs of rats without (C) or with (D) SCI. Signal intensity was analyzed at 24 hours after injection. The injection was performed at 7 days after SCI for rats with SCI. (E) The distribution ratio of FMNP-labelled U87MG was quantified and compared. Height = Average, Error bar = Standard deviation. *, *P* < 0.05.

The distribution of cells in the CNS could be different from that of Cy5.5 fluorescent dye due to their different biophysical characteristics [19]. To address these differences, U87MG glioblastoma cells were labelled with FMNP fluorescent nanoparticles (FMNP-labelled U87MG) [20, 21]. U87MG cells were utilized since their characteristics are similar with those of neural stem cells [22]. Fluorescent activities of FMNP-labelled U87MG were confirmed under a fluorescent microscope (Fig 2A). The fluorescent signal was also well detected by optical imaging in the pellet of FMNP-labelled U87MG cells (Fig 2B). FMNP-labelled U87MG cells were then injected into LVs of rats with or without SCI. The injection was performed at 7 days after SCI for rats with SCI. Motor dysfunction was confirmed by the BBB score (S1C Fig). When *in vivo* distribution of FMNP-labelled U87MG cells was analyzed by the same protocol with Cy5.5 fluorescent dye, signal intensity of FMNP-labelled U87MG cells was much weaker than that of Cy5.5 fluorescent dye (S2 Fig). To detect FMNP-labelled U87MG, the CNS including brain and spinal cord was separated at 24 hours after injection and then analyzed by optical imaging (Fig 2C and 2D).

**Fig 3:**
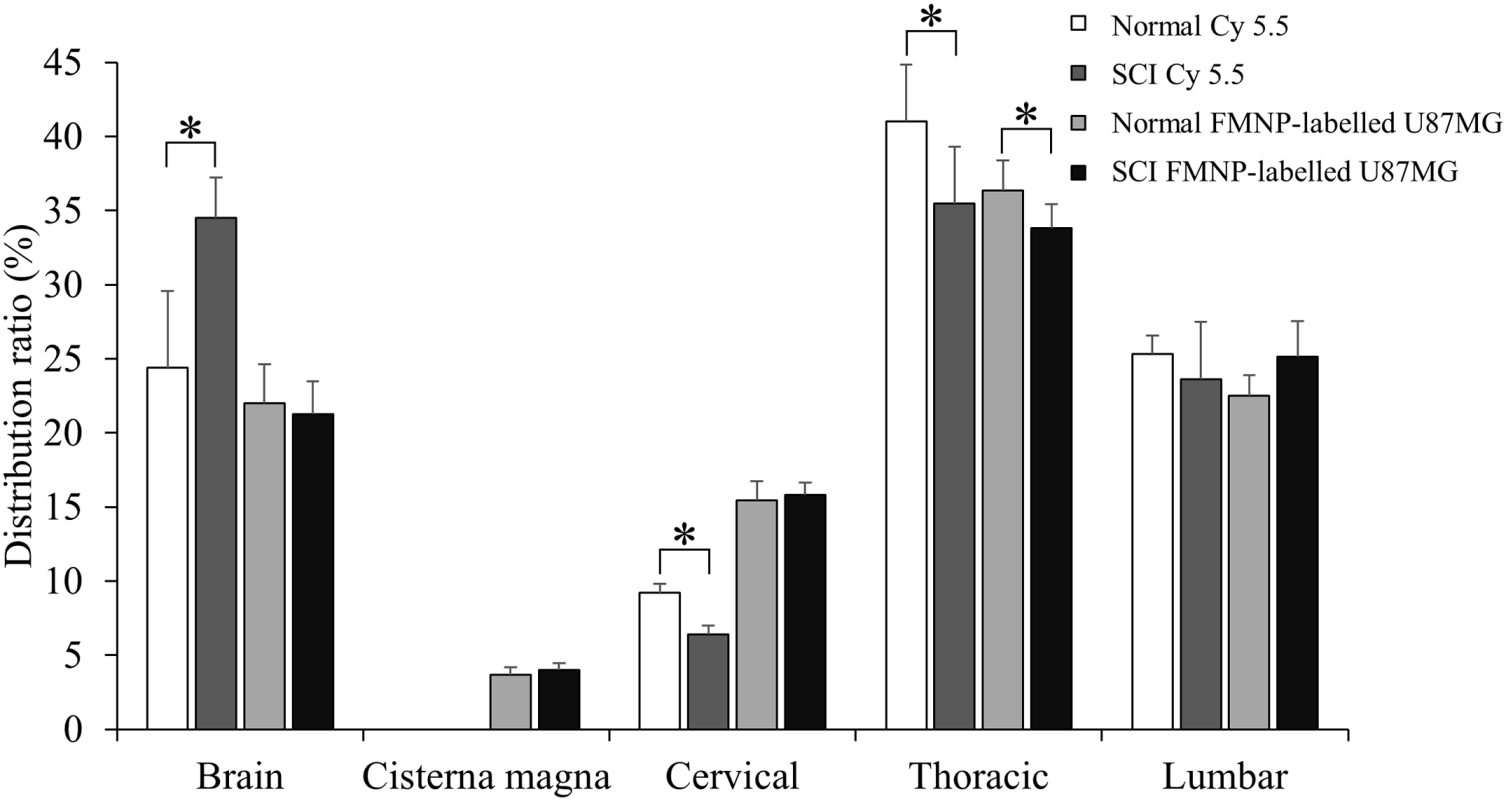
Distribution of Cy5.5 fluorescent dye and FMNP-labelled U87MG in CNS. Distribution ratios of Cy5.5 fluorescent and FMNP-labelled U87MG were compared together. Height = Average, Error bar = Standard deviation. *, *P* < 0.05.

When fluorescence signal was visualized, strong fluorescence activities of FMNP-labelled U87MG were observed from the brain to the lumbar spinal cord in both normal (Fig 2C) and SCI (Fig 2D) groups (n = 5 for each group). Quantitative analysis showed similar distribution between normal and SCI groups while signal intensity of the thoracic spinal cord in the normal group was significantly higher than that in the SCI group (Fig 2E). These results indicate that cells can also migrate well from the LV to the lower spinal cord regardless of physical injury in the thoracic spinal cord (T9). The migration of cells is also slightly reduced by SCI. When Cy5.5 fluorescent dye and FMNP-labelled U87MG signal of normal and SCI groups were compared (n = 5 for each group, Fig 3), these four groups showed similar quantitative distributions of signals. This suggests that distributions in the CNS of soluble Cy5.5 fluorescent dye and colloidal FMNP-labelled U87MG cells are similar when they are injected into CSF of the LV.

### Validation of *in vivo* distribution of U87MG cells in spinal cord

**Fig 4:**
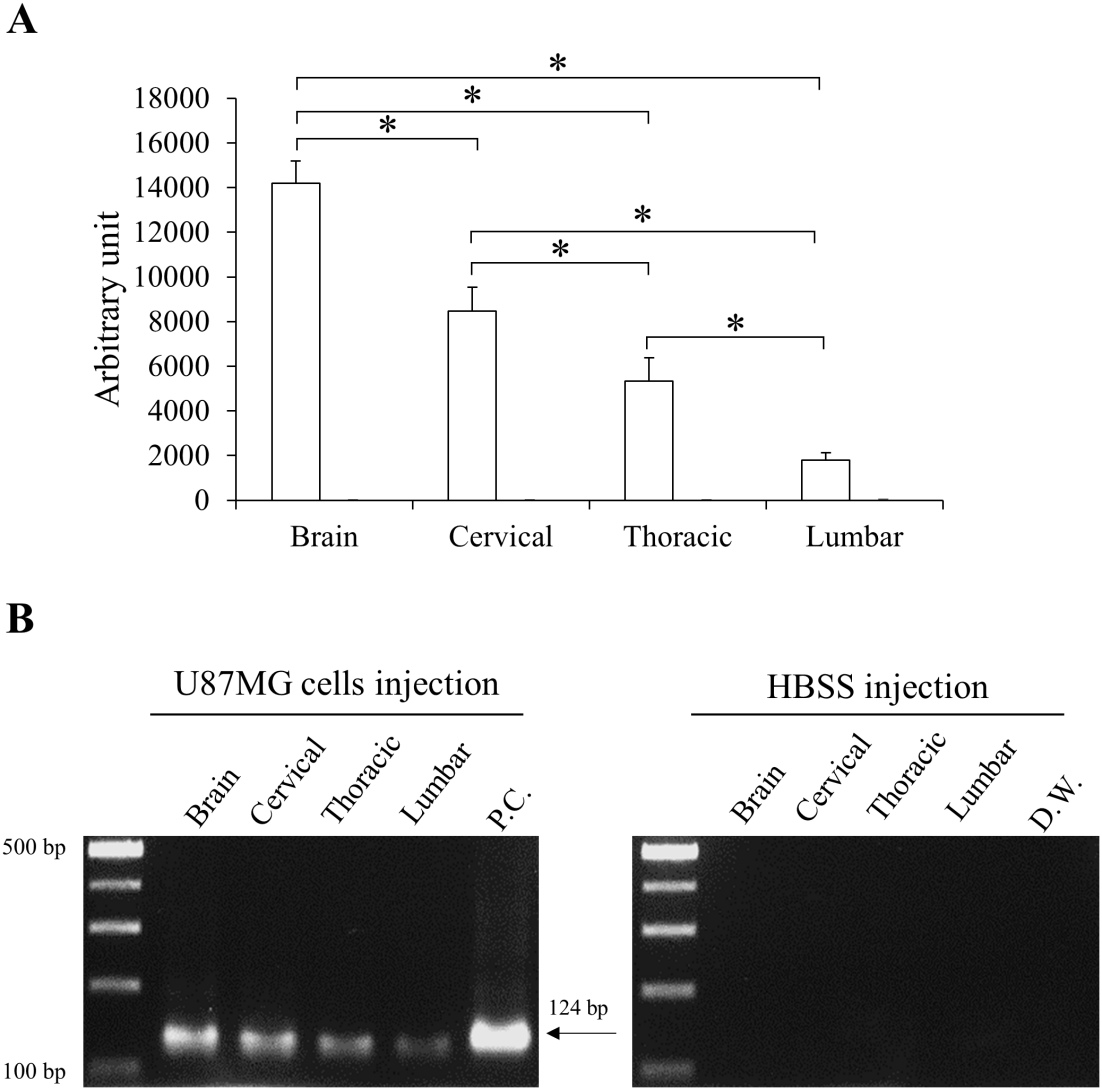
Quantitative real-time PCR (qPCR) against human specific sequence. To detect U87MGs in the CNS of rats with SCI, each region of the CNS was analyzed by qPCR using human-specific primers. (A) Relative amounts of human Alu sequence were quantified and compared. Height = Average, Error bar = Standard deviation. *, *P* < 0.05. P.C. = positive control (U87MG cells). (B) Specificity of qPCR was confirmed by the size of PCR product (124 base pairs).

**Fig 5:**
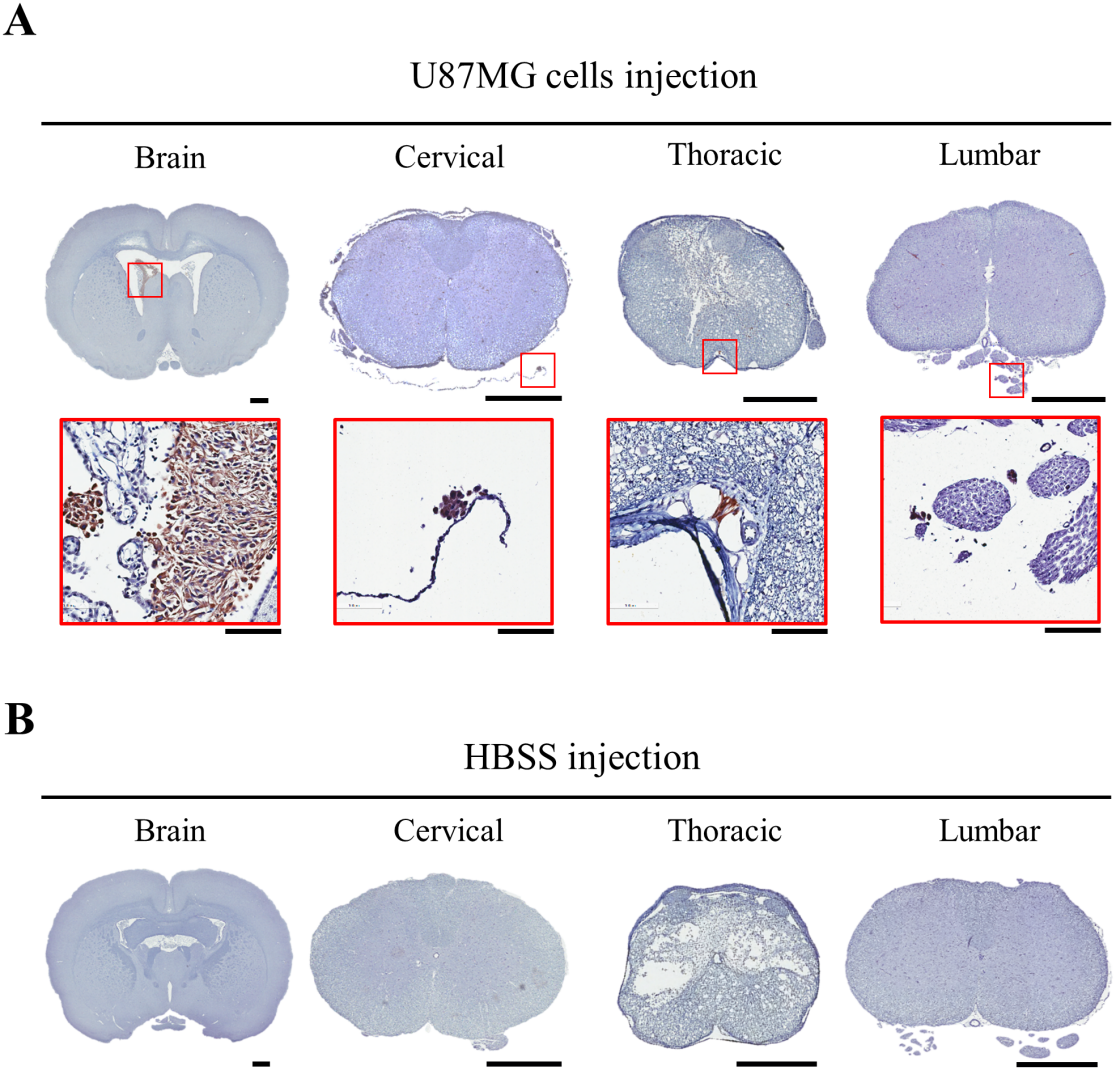
Immunohistochemical detection of human cells. Each region of the CNS in U87MG (A) or HBSS (B) group was analyzed by anti-human cytoplasm antibody. Nuclei were counter-stained by hematoxylin. Immunoreactive cells to the antibody were magnified in inlets (A). Scale bar = 1 cm for low magnification pictures and 100 μm for high magnification pictures.

The distribution of human U87MG cells in the CNS with SCI was confirmed by qPCR using human-specific Alu primers and immunohistochemistry staining using an anti-human cytoplasm antibody. U87MG cells (U87MG group) or HBSS (HBSS group) were injected to LVs of rats with SCI (n = 5 for each group) at 7 days after SCI. Motor dysfunction was confirmed by the BBB score (S1C Fig). CNSs of rats were harvested at 24 hours after the injection. In qPCR, human cells were detected in all CNS regions of rats with U87MG while the other group showed no specific reaction against human Alu gene (Fig 4). In U87MG group, relative amount of the human gene decreased significantly from the brain to the lumbar spinal cord (Fig 4A). Specificity of qPCR was confirmed by the size of PCR product (Fig 4B). When anti-human cytoplasm antibody was applied, U87MG cells were visualized in all CNS regions of the U87MG group (Fig 5A). However, no immunoreactive cells were observed in the HBSS group (Fig 5B).

## Discussion

Preclinically, numerous reports have described regenerative therapeutic effects of stem cells for neurodegenerative diseases including SCI [23]. These reports have facilitated clinical trials recently. Stem cell therapies exert their activities for SCI through several mechanisms, including rescuing injured axons, secreting neurotrophic factors, and replacing neural cells [24]. These mechanisms require stem cells in SCI lesion to mediate functions. Therefore, in clinical view, optimal dose and injection route of stem cells are important for SCI patients. To determine the optimal dose of stem cells, dose escalation studies could be performed in preclinical animal models and Phase I clinical trials. However, injection route could not be answered easily in animal models since animal models are structurally and functionally different from humans [25].

There are several possible administration routes for clinical application of stem cells to treat SCI, including intramedullary injection, intravenous injection, and intrathecal injection. Intramedullary injection into the cavity of SCI region has clinical disadvantages, including risk of unnecessary secondary injury of the spinal cord at injection site [26]. Regarding the limitation of stem cell migration through the blood-brain barrier (BBB), intravenous injection of stem cells is not promising in the CNS entry of stem cells. Intravenous injection might need to use another technique such as exogenous BBB manipulation [27]. According to reports from animal studies, intrathecal injection may be more effective in stem cell engraftment to injured site than intravenous injection [28].

CSF is watery fluid that is continuously produced in choroid plexuses of ventricles in the brain and absorbed into the venous system around the surface of the brain [29]. The mean CSF volume is 150 ml in human, including 25 ml in ventricles and 125 ml in the subarachnoid space [30]. CSF circulation from sites of production to sites of absorption largely depends on arterial pulse wave. Although CSF conversion decreases as humans grow older, CSF is renewed about four times every 24 hours in adult humans [31]. Therefore, the flow of CSF provides a fast delivery route of materials to various regions of the CNS including the spinal cord. To determine the flow of CSF, various types of substances have been used *in vivo*. Previous studies have suggested an intrathecal injection route for stem cells [32–34].

Our results are consistent with previous reports. In a previous study, we have injected Cy5.5 fluorescent dye into LV or cisterna magna of rats without SCI and found that LV injection is suitable for the delivery of Cy5.5 fluorescent dye to spinal cords by the CSF circulation [11]. In this study, we further demonstrate that LV injection is adequate for the distribution of soluble Cy5.5 fluorescent dye and colloidal FMNP-labelled U87MG cells in the entire spinal cord. We also show that LV injection is applicable to the spinal cord with physical injuries at thoracic level, although the localization of the Cy5.5 fluorescent dye and FMNP-labelled U87MG cells in the spinal cord is reduced by the injury. Our experiments had specific time points of injection of the Cy5.5 fluorescent dye and FMNP-labelled U87MG cells into the LV at 7 days after SCI with observation of their distribution in the CNS at 24 hours after the injection. The schedule was designed reflecting possible clinical trials for SCI. However, further studies with different experimental settings might be needed in the future.

In pharmacokinetic studies for stem cells, it is important to analyze stem cells in the target site of administration, which could be examined by amplification of specific sequences of stem cells, immunohistochemical detection of specific antigens of stem cells, or *in vivo* imaging [9, 35, 36]. These techniques have pros and cons. Since they have amplification steps of signals, sensitivity of detection could be superior to imaging methods. Moreover, long-term storage of specimens might be possible for experiments while *in vivo* imaging should be performed within hours after sacrifice of animals. In contrast, amplification steps might deviate from distribution ratios. Especially, amplification efficiency and specificity of detection could be influenced by initial amounts of templates and materials used in the experiments such as primers, antibodies, and enzymes [37]. Therefore, several techniques might be needed simultaneously to analyze the distribution of stem cells regarding the characteristics of each method. In this study, we used *in vivo* imaging, qPCR, and immunohistochemistry together for U87MG cells injected into LVs of rats with SCI. Discordant distribution ratios of U87MG cells might have originated from technical differences among these analysis methods.

In summary, we observed preclinical *in vivo* distribution of Cy5.5 fluorescent dye and U87MG cells after injection into LVs of rats with SCI. Our data suggest that LV injection could be translated into intrathecal administration routes for clinical trials and that cellular distribution in the spinal cord with SCI is as good as that of soluble dye in the spinal cord without SCI. These results might be applied further to the planning of optimal preclinical and clinical trials of stem cell therapeutics for SCI.

## Supporting information

S1 Fig Induction of spinal cord injury (SCI). (A) Surgical steps to make SCI are presented. Contusion injury is induced by dropping a rod of a MASCIS impactor onto the T9 spinal cord (right) that is exposed by laminectomy (left). (B) After SCI, functional disability of hindlimb is confirmed on open field. (C) Functional effects of SCI are quantified by Basso, Beattie and Bresnahan (BBB) test at 7 days after SCI. normal, n = 10; SCI, n = 20. Height = Average, Error bar = Standard deviation. *, *P* < 0.05.
(TIF)

S2 Fig Comparison of *in vivo* signal between Cy5.5 injection group and FNMP-labelled U87MG injection group. Cy5.5 fluorescent signal and FMNP-labelled U87MG was observed by *in vivo* optical imaging at 24 hours after injection. 20nM Cy5.5 fluorescent dye or 5 × 10^6^ FMNP-labelled U87MG in HBSS was injected into the lateral ventricle at 7 days after SCI. H = Head, C = Cervical, T = Thoracic, L = Lumbar.
(TIF)
