# Supplementary figures and images for: In vivo distribution of U87MG cells injected into the lateral ventricle of rats with spinal cord injury
Source: PLoS One. 2018 Aug 16;13(8):e0202307. doi: 10.1371/journal.pone.0202307 (PMC6095526; doi:10.1371/journal.pone.0202307)

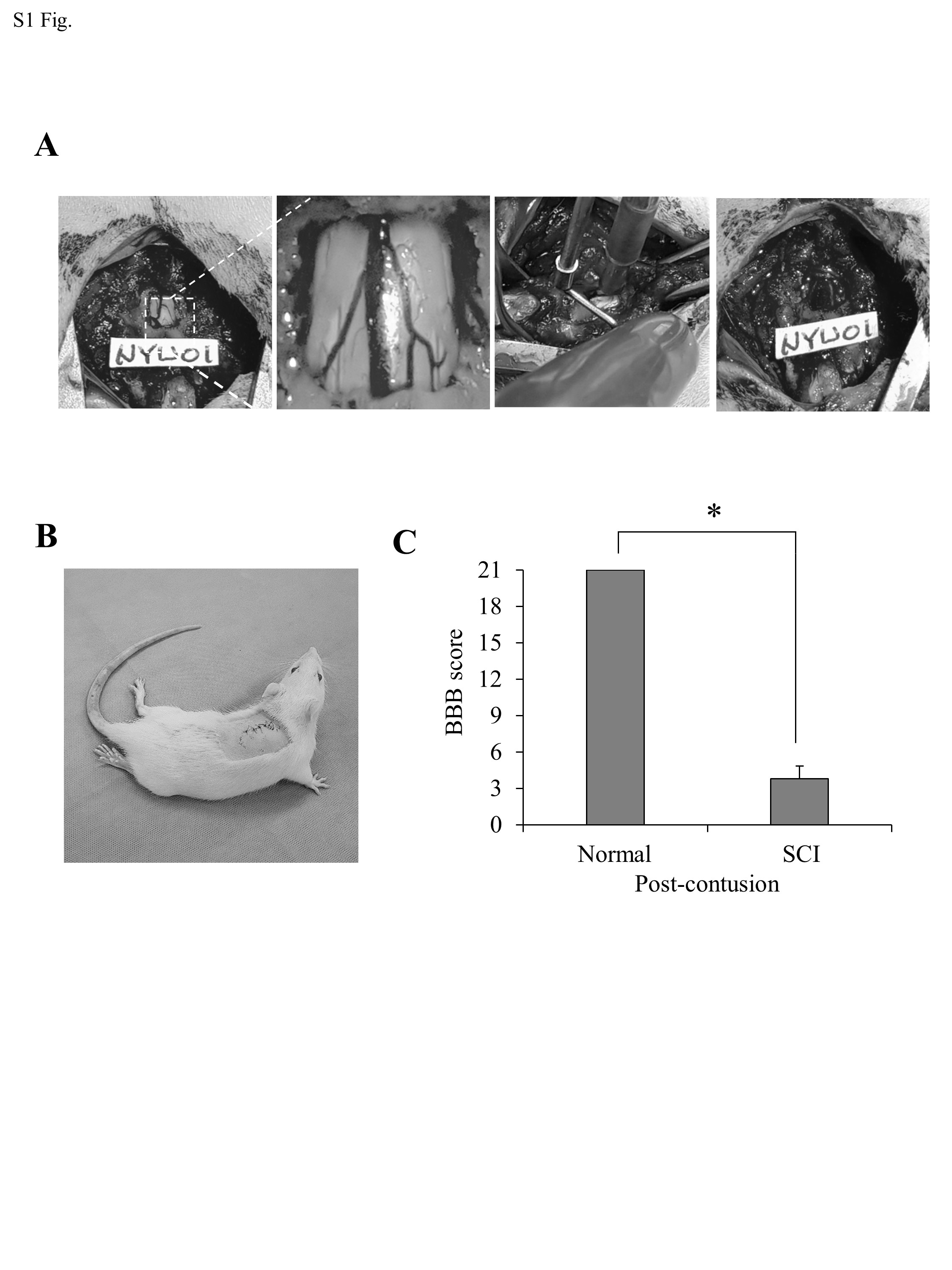

Supplement: S1 Fig — (A) Surgical steps to make SCI are presented. Contusion injury is induced by dropping a rod of a MASCIS impactor onto the T9 spinal cord (right) that is exposed by laminectomy (left). (B) After SCI, functional disability of hindlimb is confirmed on open field. (C) Functional effects of SCI are quantified by Basso, Beattie and Bresnahan (BBB) test at 7 days after SCI. normal, n = 10; SCI, n = 20. Height = Average, Error bar = Standard deviation. *, P < 0.05. (TIF) [file pone.0202307.s001.TIF]

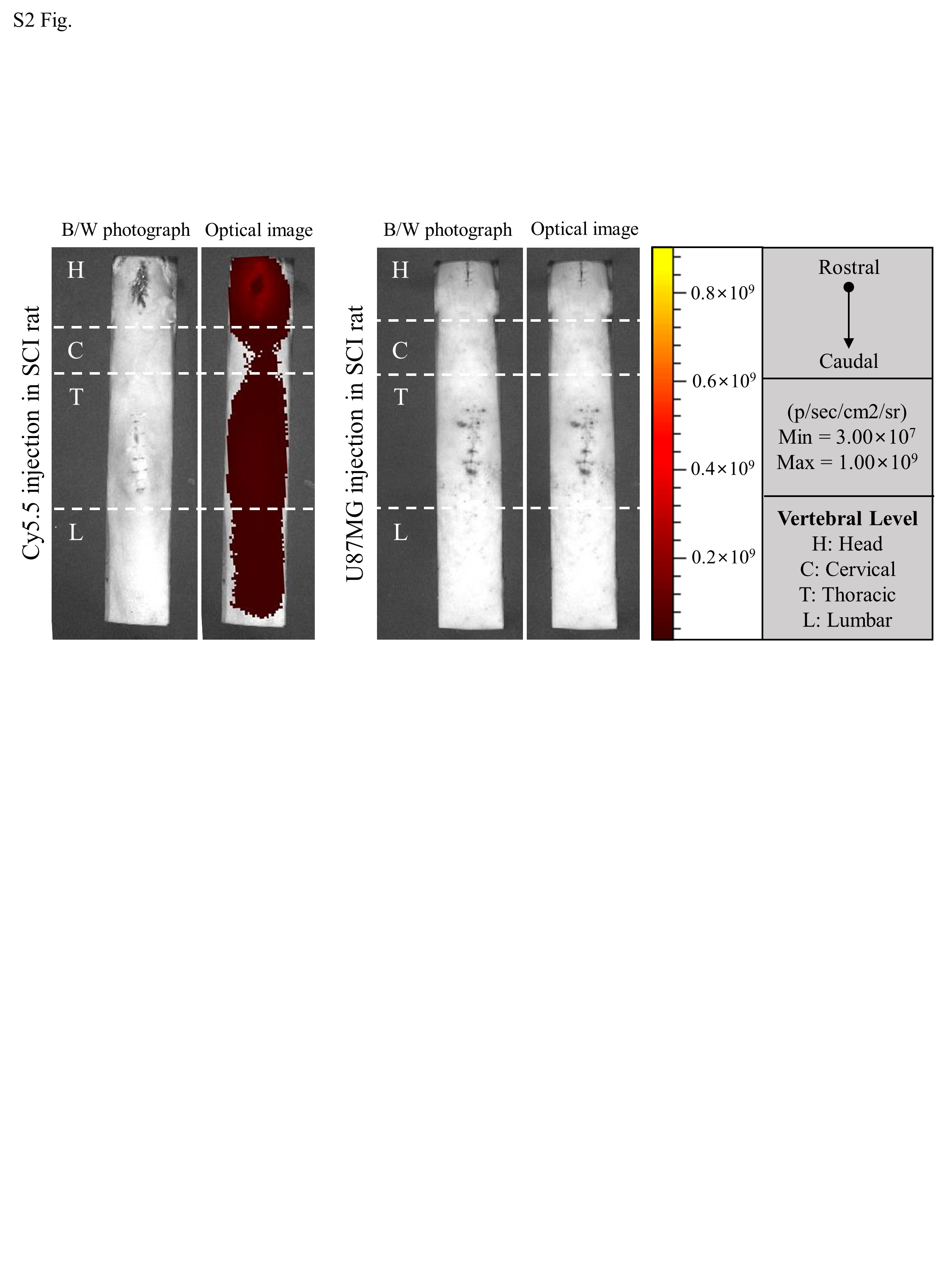

Supplement: S2 Fig — Cy5.5 fluorescent signal and FMNP-labelled U87MG was observed by in vivo optical imaging at 24 hours after injection. 20nM Cy5.5 fluorescent dye or 5 × 10^6^ FMNP-labelled U87MG in HBSS was injected into the lateral ventricle at 7 days after SCI. H = Head, C = Cervical, T = Thoracic, L = Lumbar. (TIF) [file pone.0202307.s002.TIF]
